# Reasons for non-vaccination against influenza among older adults with hypertension in Brazil: a cross-sectional study

**DOI:** 10.1590/1516-3180.2020.0042.R1.15052020

**Published:** 2020-07-03

**Authors:** Aldiane Gomes de Macedo Bacurau, Priscila Maria Stolses Bergamo Francisco

**Affiliations:** I MSc. Nurse and Postgraduate Student (doctoral), Department of Collective Health, Faculdade de Ciências Médicas (FCM), Universidade Estadual de Campinas (UNICAMP), Campinas (SP), Brazil.; II PhD. Statistician and Associate Professor, Department of Collective Health, Faculdade de Ciências Médicas (FCM), Universidade Estadual de Campinas (UNICAMP), Campinas (SP), Brazil.

**Keywords:** Aged, Influenza vaccines, Hypertension, Health surveys, Vaccination, Chronic disease, Elderly, Disease prevention, High blood pressures, Awareness, vaccination

## Abstract

The aim of this study was to estimate the prevalence of non-vaccination against influenza among Brazilian older adults with systemic arterial hypertension and determine the main reasons for non-adherence. A cross-sectional study was conducted using data from older adults (≥ 60 years of age) with hypertension who participated in the 2013 National Health Survey and reported not having been vaccinated against flu over the previous 12 months (n = 1,295). The analyses were performed using the Stata 14.0 software. The data were weighted because of the sampling design. An estimated 3,026,080 older adults with hypertension had not received a flu vaccine over the 12 months prior to the survey (22.6%). No significant associations were found with sex, age group or schooling. The prevalence of unvaccinated older adults was lower in the southern and southeastern regions of Brazil than in the northern and northeastern regions, even after adjusting for age. The prevalence was higher among individuals without private health insurance. The main reasons for non-vaccination were *fear of a reaction, rarely having the flu and not believing in the protection of the vaccine*. The present findings underscore the need for healthcare professionals to explain to the population the benefits of the vaccine for preventing severe influenza (protective effect and possible reactions) and for secondary prevention of cardiovascular events. Increasing the prevalence of vaccination in older adults with hypertension and other cardiovascular diseases is of fundamental importance within the realm of public health as a strategy for reducing occurrences of complications and deaths associated with influenza.

## INTRODUCTION

Systemic arterial hypertension is a major risk factor for other cardiovascular diseases^1^ and is highly prevalent in both adults and elderly people.^2^^,^^3^ Data from the Brazilian National Health Survey revealed rates of 44.4%, 52.7% and 55.0% among Brazilian elderly people aged 60-64, 65-74 and ≥ 75 years, respectively,^4^ and the prevalence increased with age (71.7% of individuals aged ≥ 70 years had high blood pressure or reported taking antihypertensive medication).^3^

Individuals with cardiovascular disease are at greater risk of complications from influenza.^5^^,^^6^ Besides the risk factors described in the literature (hypertension, obesity, physical inactivity, smoking, etc.), influenza contributes to cardiovascular morbidity and mortality.^6^ The American Heart Association and American College of Cardiology indicate the flu vaccine for individuals with atherosclerotic disease.^7^ The United Kingdom National Clinical Guideline Centre^8^ and the Brazilian Cardiology Society^9^ indicate the vaccine for individuals with heart failure.

Studies have shown that in individuals with cardiovascular disease, the flu vaccine reduces occurrences of cardiovascular events and mortality.^6^^,^^10^ Among individuals with hypertension, vaccination prior to the flu season has been significantly associated with reduction of the risk of death due to acute myocardial infarction, stroke and all causes.^10^

In Brazil, the flu vaccine is offered through the public healthcare system to groups that are at risk (elderly people and individuals with chronic respiratory, heart, neurological, liver, kidney and metabolic diseases), as a strategy for prevention of the disease, its severe forms and complications.^11^ Higher rates of vaccination among individuals with hypertension have been observed since these campaigns began.^12^^,^^13^

Since hypertension is a chronic disease that requires follow-up and treatment, most older adults in Brazil are dependent on the public healthcare system^14^ and primary care is the main source of antihypertensive medications.^15^ Thus, it can be hypothesized that this group is more attentive to information on vaccination campaigns and other offers from public healthcare services. However, after two decades of vaccine campaigns, approximately 20% of elderly people with hypertension are not receiving the vaccine, and this percentage has remained stable over the years.^12^^,^^16^

## OBJECTIVE

The aim of the present study was to estimate the prevalence of non-vaccination against influenza among older Brazilians with hypertension and determine the main reasons for non-adherence.

## METHODS

A cross-sectional study was conducted using public domain data on elderly people (≥ 60 years) who participated in the 2013 National Health Survey,^17^ reported having hypertension (n = 5,524) and reported having not been vaccinated against influenza over the previous 12 months (n = 1,295). We estimated the absolute number and proportion of non-vaccinated elderly people with hypertension and the respective 95% confidence intervals, according to sociodemographic characteristics, and determined the reasons for non-vaccination.

All estimates were made using the Stata 14.0 software and took the sampling design into consideration. The National Health Survey had received approval from the National Ethics Committee of the Health Ministry (certificate number: 328.159; June 26, 2013).

## RESULTS

The mean age of the elderly people with hypertension was 70.3 years (95% confidence interval, CI: 70.0-70.7), and 61.0% (95% CI: 58.8-63.2) were women. It was estimated that 3,026,080 elderly people with hypertension had not been vaccinated against influenza (22.6%; 95% CI: 20.9-24.5). No significant associations were found in relation to sex (P = 0.373), age group (P = 0.456) or schooling (P = 0.138). In comparison with the northern region of the country (27.9% not vaccinated), the prevalence of non-vaccinated elderly people was lower in the southern and southeastern regions (16.7% and 20.8%, respectively), even after adjusting for age. The prevalence of non-vaccination was higher among individuals without private health insurance P = 0.026 (Table 1).

**Table 1.** Vaccination against influenza among older Brazilians with hypertension, according to sociodemographic characteristics. National Health Survey, 2013

Variables	n	Unvaccinated		Vaccinated	
		%	95% CI	%	95% CI
Region					
North	751	27.9	22.0-34.6	72.1	65.4-78.0
Northeast	1,620	29.0	25.7-32.5	71.0	67.5-74.3
Center-West	653	25.1	20.5-30.4	74.9	69.6-79.5
Southeast	1,659	20.8	18.1-23.8	79.2	76.2-81.9
South	841	16.7	13.5-20.4	83.3	79.6-86.4
Sex					
Male	1,908	21.5	18.7-24.7	78.5	75.3-81.3
Female	3,616	23.3	21.1-25.7	76.7	74.2-78.9
Age group					
60 to 69	2,917	22.0	19.8-24.3	78.0	75.6-80.2
70 to 79	1,810	23.6	20.4-27.2	76.4	72.8-79.6
80 or more	797	22.8	18.0-28.5	77.2	71.5-82.0
Race/skin color					
White	2,609	21.2	18.7-24.0	78.8	76.0-81.3
Nonwhite	2,914	24.2	21.8-26.8	75.8	73.2-78.2
Lives with spouse/partner					
Yes	2,453	20.4	17.7-23.3	79.6	76.7-82.2
No	3,071	25.5	23.0-28.2	74.4	71.8-77.0
Schooling					
None/incomplete primary school	3,977	23.5	21.3-25.7	76.5	74.3-78.6
Complete primary and high school	1,040	20.6	17.0-24.7	79.4	75.3-83.0
Incomplete/complete university	507	19.8	14.5-26.4	80.2	73.6-85.5
Can read and write					
Yes	4,114	21.3	19.3-23.5	78.7	76.5-80.7
No	1,410	27.1	23.5-31.0	72.9	69.0-76.5
Private health insurance					
Yes	1,676	19.7	16.8-22.9	80.3	77.1-83.2
No	3,848	24.0	21.9-26.3	76.0	73.7-78.1

CI = confidence interval (α = 0.05), considering the study design effect.

**Figure 1.**
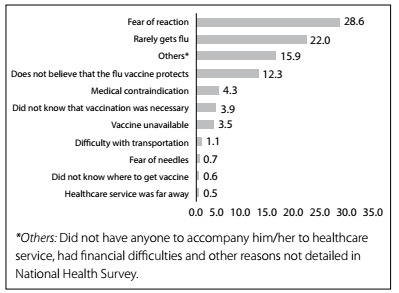
Distribution of reasons for non-adherence to vaccination against influenza among elderly people with hypertension. National Health Survey, 2013.

The main reasons for non-vaccination were *fear of a reaction* (28.6%; 95% CI: 24.9-32.6)*, rarely having the flu* (22.0%; 95% CI: 18.9-25.4) and *not believing in the protection of the vaccine* (12.3%; 95% CI: 9.5-15.8) (Figure 1).

The prevalence of non-vaccination among elderly people with hypertension was lower than the rates found for elderly people in general and for those who reported not having hypertension (27.4% and 31.9%, respectively).

## DISCUSSION

In the present study, the prevalence of vaccination against influenza among elderly people with hypertension was lower than what was expected for the general population of elderly people, given that the goal in 2013 was to vaccinate at least 80% of all individuals ≥ 60 years of age.^11^ Considering the greater contact of this group with healthcare services,^12^^,^^15^ the absolute number of unvaccinated individuals was high. Previous studies also found that there were no associations between the vaccination rate and sex,^12^^,^^16^^,^^18^^,^^19^ age group^18^ or schooling.^18^^,^^19^ Sato et al.^19^ found that the chance of having been vaccinated was greater among elderly people registered with the Family Health Program.

Regarding regional differences, the southern and southeastern regions of Brazil present socioeconomic differences in relation to the northeastern region. This may be reflected in access to healthcare services and, consequently, to information and counseling regarding the importance of vaccination. Moreover, the seasonality of influenza is more pronounced in the more southerly regions. In contrast, in northeastern Brazil, the peaks of the disease occur prior to the period when vaccination campaigns have been run,^20^ and this may have had an impact on the effectiveness of such campaigns as well as on the perceptions of elderly people regarding the protection offered by the vaccine, which thus will have had a negative influence on adherence.

The reasons for non-vaccination given by these elderly people with hypertension were similar to those found for the older population in general.^18^^,^^19^ Fear of side effects falsely attributed to the vaccine, not considering it important and having insufficient information regarding the benefits were the main reasons given.^12^^,^^21^ Counseling by healthcare professionals has been positively associated with vaccination and should be used as a strategy for improving knowledge among elderly people regarding both the disease and the vaccine.

## CONCLUSION

The main reasons for non-vaccination (fear of a reaction, belief that influenza is a rare event, belief that the vaccine does not offer protection and fear of needles) accounted for more than 60% of the reasons given by these elderly people. These findings underscore the need for health professionals to explain to the population what the benefits of the vaccine are, regarding prevention of severe influenza (its protective effect and possible reactions) and secondary prevention of cardiovascular events. Increasing the prevalence of vaccination among elderly people with hypertension and other cardiovascular diseases is of fundamental importance within the realm of public health, as a strategy for reducing occurrences of complications and deaths associated with infection by the influenza virus.
